# Unveiling Intraprofessional Dynamics: Learning Teamwork in Acute Care Consultations Between Paediatric and General Practice Residents

**DOI:** 10.5334/pme.1770

**Published:** 2025-11-13

**Authors:** Rosalin M. van Schie, Ester H. A. J. Coolen, Iris Schoorlemmer, Nina R. Aalfs, Janielle A. E. M. van der Velden, Nynke D. Scherpbier-de Haan, Bart P. A. Thoonen

**Affiliations:** 1 Department of Primary and Community Care, Radboud University Medical Centre, Nijmegen, The Netherlands; 2 Department of Paediatrics, Radboud University Medical Center Amalia Children’s Hospital, Nijmegen, The Netherlands; 3 Primary and Long-term Care, University Medical Centre Groningen, Groningen University, Groningen, The Netherlands; 4 Development of Education Primary Care, Department of Primary and Community Care, Radboud University Medical Centre, The Netherlands

## Abstract

**Introduction:**

Effective remote collaboration under high-pressure conditions—such as physicians consulting each other by phone about acutely ill patients—is a complex aspect of teamwork. Despite its importance for patient outcomes, medical training has often overlooked the emotional and cognitive dynamics that shape teamwork. To address this, we developed an intraprofessional consultation training (IPCT) for paediatric (P) and general practice (GP) residents, based on the Teamwork-Attitude, Behaviour, Cognition (ABC) model, to enhance teamwork skills in acute care. This study examined what residents learned about consultation dynamics, their intended behavioural changes, and how these translated into practice.

**Methods:**

This qualitative study involved GP and P residents from the Radboud University Medical Centre, Nijmegen, the Netherlands and three affiliated non-academic teaching hospitals. The IPCT included video reviews, reflections, and role-playing. Residents completed reflection forms and set intentions. Two months later, a follow-up questionnaire was administered. Template analysis, guided by the “ABCs of teamwork” framework, was conducted. Learning outcomes and transfer were assessed using the Kirkpatrick model.

**Results:**

Twenty-six residents (12 GPs, 14 Ps) participated. The IPCT fostered shared mental models and alignment. Residents primarily gained insight into the attitudes and emotions shaping teamwork, becoming more aware of their own and others’ emotions and interactional dynamics. Although they applied these insights in practice, managing complex intraprofessional dynamics remained challenging.

**Conclusion:**

Residents developed strategies to navigate emotional dynamics of teamwork, aligning with the concept of mentalising—self-reflection, empathy, and relational interpretation. Yet applying these strategies in clinical practice remained challenging within the existing high-pressure medical work culture and warrants attention.

## Introduction

Delivering high-quality patient care requires precise alignment and coordination from all healthcare professionals involved. Achieving such collaboration in practice, however, remains challenging [1]. A considerable number of adverse events in medical settings stem from communication breakdowns among healthcare professionals [2,3,4,5,6]. One critical yet sensitive aspect of collaboration is intraprofessional consultation, which refers to consultations among professionals within the same discipline. In the context of physician intraprofessional consultation, this involves collaborating to share expertise on a patient’s condition and to determine the most appropriate course of treatment [4,6]. Such consultations require the co-construction of a shared mental model, conceptualised as a common understanding and approach to an acutely ill patient by physicians entering with differing initial perspectives [7]. In acute care, the complexity of intraprofessional consultations increases when physicians collaborate remotely, often by phone and synchronously, but from different care contexts— rather than in person [2]. Working ad hoc across clinical contexts to manage an acutely ill patient heightens the urgency for swift decision-making, adding to the pressure. These aspects make intraprofessional consultation in acute care a highly complex form of team collaboration [8].

Within team collaboration, members’ collaborative behaviours can be categorised into taskwork and teamwork [9,10,11]. Taskwork pertains to ‘what’ is done, involving the execution of core medical competencies, whereas teamwork refers to ‘how’ it is done, encompassing the range of interactive behavioural processes. Effective teamwork is just as crucial as effective taskwork for achieving a successful collective patient outcome [9,10]. Effective teamwork requires cohesive team dynamics—an often invisible process in which mutual trust, understanding, and respect are crucial [12]. This requires reflection from healthcare professionals on their attitudes and cognitions toward themselves and others in order to gain understanding and control over these underlying processes [13].

Remarkably, physicians receive minimal training in recognising, acknowledging, and managing the underlying processes that underpin effective collaborative behaviour, leaving these dynamics to be learned unintentionally [5,8,14,15,16,17,18]. This highlights a critical gap in medical education, particularly in the postgraduate phase, underscoring the importance of fostering teamwork competencies to bridge the gap between siloed educational environments and the collaborative demands of clinical practice. This is especially crucial for physicians training in remote settings, who must collaborate effectively across diverse contexts under pressure, such as paediatric and general practice residents, particularly when managing the complex care of acutely ill children [19,20].

Therefore, we developed and evaluated an intraprofessional consultation training (IPCT) for paediatric (P) and general practice (GP) residents. This IPCT focused on the underlying dynamics of challenging intraprofessional acute care consultations, based on the “teamwork ABC” framework, which defines teamwork as “a set of interrelated cognitions (C), attitudes (A), and behaviours (B) contributing to the dynamic processes of team performance” [21,22,23].

The aim of this study was to investigate what P and GP residents learned about the influence of dynamics on consultation behaviour when managing acutely ill children, what behavioural changes they intended to apply in clinical practice, and how these insights and intentions were transferred into practice two months post-training.

## Methods

### Research context

#### Population

The study population consisted of GP residents from the Radboud University Medical Centre (Radboudumc) Nijmegen, the Netherlands, and P residents from the affiliated teaching hospitals Canisius Wilhelmina Hospital (CWZ) in Nijmegen, Rijnstate Hospital in Arnhem, and Jeroen Bosch Hospital (JBZ) in ‘s-Hertogenbosch, the Netherlands.

#### Context

We developed an IPCT for P and GP residents who had previously enrolled in an intraprofessional educational programme focusing on collaborative care for acutely ill children [23]. In this programme, P and GP residents jointly assess acutely ill children in each other’s context and attend educational sessions on collaborative child care. The IPCT designed for this study was incorporated into one of these educational sessions.

### Research design

Template analysis was applied to examine what P and GP residents learned about the influence and interrelationship between teamwork attitudes (A), cognitions (C), and behaviours (B), and how they applied insights and behavioural intentions from the IPCT in clinical practice two months post-training [24]. The comprehensive “teamwork ABC” framework served as an initial template, as outlined in Appendix A [21,24]. To assess learning outcomes and transfer these into practice, we additionally used the Kirkpatrick model [25].

### Framework

**Figure 1:**
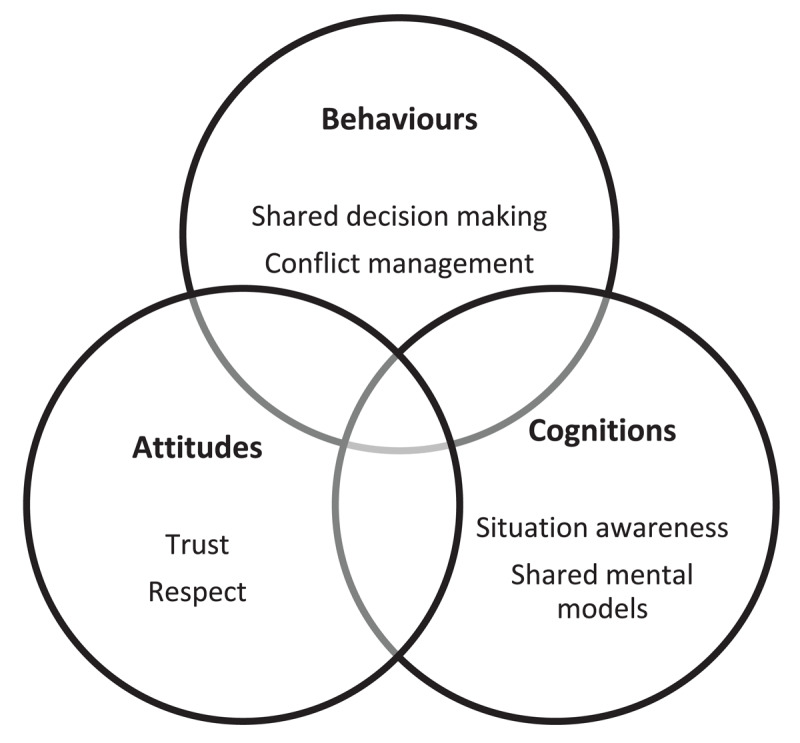
Comprehensive framework of the teamwork ABC in healthcare with ABC examples [21].

The “teamwork ABC” defines teamwork as a dynamic process driven by the interconnected affective and motivational states, behavioural processes, and cognitive states (Figure 1) [21]. The attitudes (A) reflect the team’s emotions and motivations towards both the task and teammates (e.g., mood, trust). Cognitions (C) encompass the structure of knowledge, information acquisition, shared perceptions, and task allocation (e.g., shared mental models). Behaviours (B) encompass the actions that teams undertake (e.g., conflict management). The ABCs of teamwork influence one another recursively and become increasingly important as intrateam interdependence intensifies [22].

### Design of the intraprofessional consultation training (IPCT)

#### IPCT instructors and research team

The IPCT was conducted by a fixed team of GP and behavioural science instructors from the Department of Primary Care Medicine at the Radboud University Medical Centre. Instructors did not participate in the research team.

#### IPCT design

**Figure 2:**
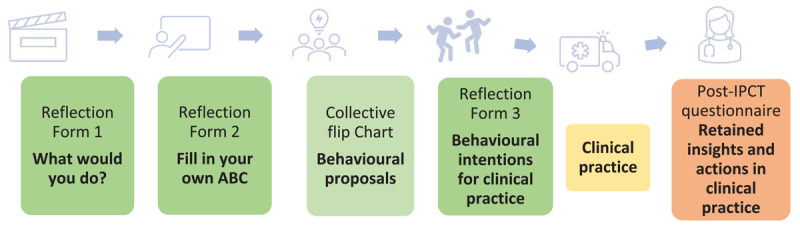
Design of the IPCT and data collection within and after IPCT. During the training, three individual reflection forms were completed, and a flip chart was collectively filled out (green). Two months post-training, insights retained and transferred to clinical practice were evaluated through a post-IPCT questionnaire (orange).

The IPCT was based on the ABC framework and was designed by the research team (RS, EC, NA, JV, NS, BT), drawing on experiential learning principles and phases (Figure 2) [26]. The IPCT began with a review of a video showing a challenging intraprofessional consultation between a GP and a P regarding a potentially acutely ill child, performed by trained actors (Appendix B, video scenario). Following the video, residents individually completed Reflection Form 1, documenting the actions they would take during the consultation. Subsequently, the ABC model of intraprofessional dynamics was explained, after which the video was reviewed. Residents completed Form 2, describing their ABCs, followed by a group reflection in which residents exchanged their ABCs and described effective behaviours on flip charts. Next, pairs of residents role-played the scenario, incorporating their proposed behaviours. During the exercise, residents responded to each ‘other’s behaviour by adjusting their physical proximity to convey emotional distance or closeness. With instructor support, residents reflected on which behaviours created feelings of distance or closeness and how these influenced the consultation. The IPCT concluded with an individual reflection session, in which residents documented two concrete intentions for their clinical practice on Form 3.

Two months post-IPCT, follow-up questionnaires were administered to evaluate retained insights and application of intentions in practice (Appendix C). This open-ended questionnaire was based on the ABC framework and the Kirkpatrick model.

### Data collection

The completed training forms and flip charts were collected at the end of each IPCT. Form 3, documenting intended behavioural changes in intraprofessional consultation, was photographed by the participants for future reference. Post-IPCT questionnaires were sent to the participants two months following the IPCT. The completed forms, flip charts, and questionnaires were pseudonymised by RS.

### Data analysis

Data were analysed by RS and IS using Atlas.ti version 24.0.0 software. The ABC framework served as the foundation for the initial coding template, incorporating seven a priori themes (Appendix A) [24]. Given the central role of teamwork behaviour, behavioural intention, and behaviour change in our initial template, IS and RS predefined these concepts to ensure intercoder reliability and minimise divergent interpretations (see Definitions box). RS and IS independently coded the data, made descriptive notes, and progressively refined the coding template through ongoing discussions. Through systematic collaborative rereading and discussion of the data, codes were defined, reviewed with BT and EC, and refined until consensus was reached. Throughout the analysis, emerging themes were systematically discussed with the research team (EC, BT, NA, JV, NS) to achieve consensus and ensure a shared understanding. The analysis was finalised when the research team agreed that the final template encompassed all identified topics and reflected all key insights, behavioural intentions, and changes.

**Definition box predefined definitions used in the data analysis**

**DEFINITIONS**

**Table:** 

**Teamwork behaviour**	The actions individuals undertake in a team setting, categorised into “attitude-driven behaviour” and “cognition-driven behaviour”.
**Attitude-driven behaviour**	Behaviour in which feelings, perceptions, and principles about oneself and/or relationships with others are acknowledged, expressed, or discussed.
**Cognition-driven behaviour**	Behaviour based on cognitive processes involved in acquiring, processing, and structuring (medical) knowledge and information.
**Behavioural intention**	Intended changes that residents planned to make in clinical practice.
**Behavioural change**	Teamwork behaviours that residents actually adopted two months after the IPCT.

### Reflexivity and the research team

The research team consisted of general practitioners (RS, BT, NS), paediatricians (EC, JV), educationalists (NA, BT), and a 5th-year medical student (IS), ensuring a diversity of perspectives. RS, NA, and IS were actively engaged as observers of the IPCT and in conducting research and analysis, while EC, BT, JV, and NS contributed to data interpretation and reflection without being involved in the IPCT. As programme directors of the paediatric residency programme, EC and JV were not present during the IPCT sessions. Furthermore, neither the IPCT instructors nor the observing researchers at the IPCT were involved in the ‘residents’ postgraduate training or assessments, ensuring a safe and independent learning environment for the residents. The diverse team composition fostered open discussions, integrating clinical experiences and assumptions to enhance intersubjectivity and confirmability.

### Ethics

This study was reviewed and approved by the NVMO (Netherlands Association for Medical Education) Ethical Review Board (NERB dossier number 2023.5.1). Written informed consent was obtained before participation.

## Results

### Participants and response

Twenty-six residents (12 GPs and 14 Ps) participated in this study. Three IPCT sessions took place between July and October 2023. In total, 78 forms and 6 flip charts were analysed. Subsequently, post-training questionnaires were analysed two months later, with a response rate of 92% (24 out of 26; 11 GPs and 13 Ps).

### Training and Clinical Practice: ‘Residents’ Development, Intentions and Actions

Below we provide an overview of the results in two parts. The first part describes ‘residents’ development and experiences during the training. At the start of the training, ‘residents’ reflections were elicited after viewing a consultation video between a GP and a paediatrician for the first time, and after receiving theoretical input on the Teamwork ABC followed by re-watching the video. Mid-training, behavioural proposals were formulated based on group reflections. At the end of the training, residents described the individual intentions they set for medical practice after completing the exercise, which were categorised into overarching themes.

The second part, focusing on medical practice—from intention to action and the challenges to applying intentions in practice—describes how these intentions were translated into clinical practice and the barriers encountered.

### Part 1: Development of Teamwork ABC Experiences in Training

#### Start of Training: *Behavioural strategies emerging from individual reflections*

We found that, at the start of the training, teamwork behaviours (B) were primarily cognitively (C) focused, emphasising the clarification of medical information and the restructuring of handover processes. Residents focused on identifying the requirements needed to gain a comprehensive overview of the situation.

*Start with a clear request: “I would like you to assess this child”*.

*Provide the vital signs*.

(FC8 GP resident Reflection form 1 what would you do?)

*Ask about red flag symptoms*.

*Ask early about the main reason for referral*.

(FJ P3 resident Reflection Form 1 what would you do?)

Attitude-driven behavioural proposals (A) were less prominent and primarily reflected residents’ beliefs about the attitude they should adopt during challenging consultations. They emphasised the importance of maintaining a “neutral” stance, even when experiencing irritation, uncertainty, or unrest.

*Try to avoid discussion*.

(FR GP1 resident Reflection Form 1 what would you do?)

*I would not mention being busy to the GP, I would not interrupt midway*.

(FR P5 resident Reflection Form 1 what would you do?)

**Figure 3:**
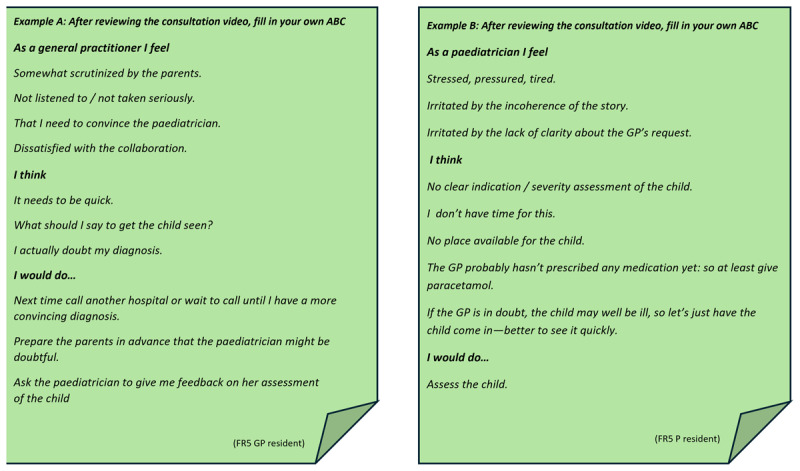
Examples from Reflection Form 2. Example A shows the feeling, thoughts and intended actions (ABC) of a GP resident, and Example B shows that of a paediatric resident.

As the training progressed, residents completed the second individual reflection form following theoretical input and a review of the consultation video, in which they described their own feelings, thoughts, and intended actions. These reflections revealed substantial internal cognitive and emotional responses. Translating these inner experiences into overt collaborative behaviour proved difficult, as illustrated by examples from a GP and a P resident (Figure 3). The feelings and thoughts the GP and P resident described—such as stress, uncertainty, unclear understanding of the ‘child’s condition, or dissatisfaction with collaboration—were minimally reflected in the actions they indicated they would take. This highlights the discrepancies between ‘residents’ internal experiences and their outward expressions, with a tendency toward avoidance, either by leaving issues unaddressed or redirecting their actions.

#### Mid-Training: *Behavioural proposals following group reflections*

**Figure 4:**
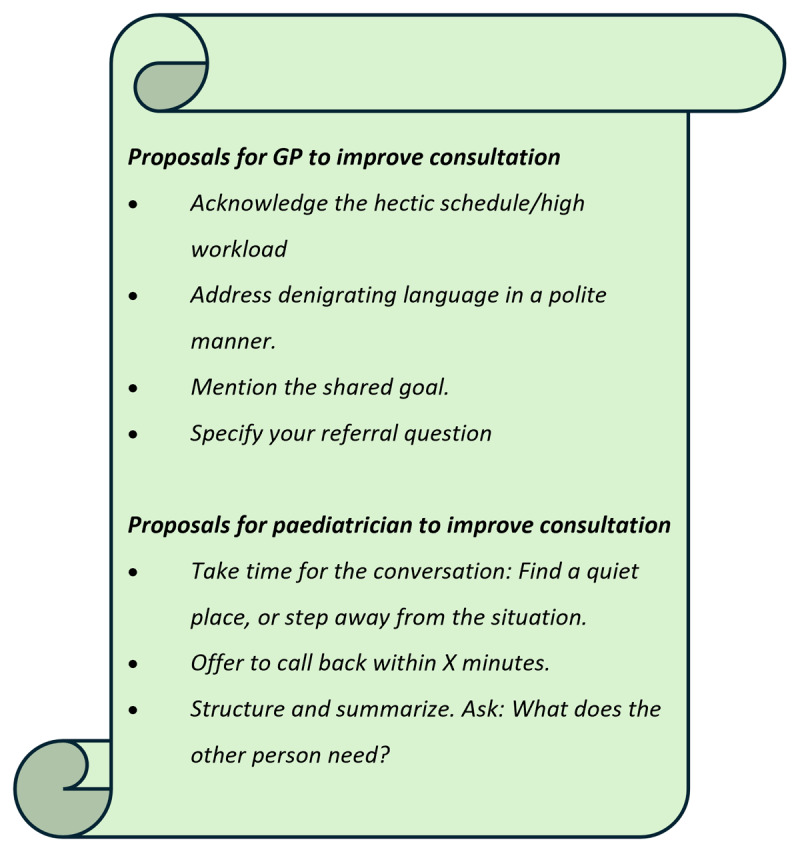
Flipchart (RSG1) example with behavioural proposals from a mixed subgroup of GP and P residents after group reflection.

Following group reflections, in which individual insights were shared and discussed, behavioural proposals were documented on flipcharts. These proposals suggested an increased consideration of ‘residents’ own feelings, the perspective of the other professional, and the shared goal as illustrated by an example flipchart from one of the subgroups (Figure 4). The flipchart included strategies such as acknowledging stress and time pressure rather than ignoring them, and arranging practical solutions like call-backs. It also emphasised actively clarifying the ‘other’s needs rather than assuming them, and addressing irritations in the tone of collaborative conversations (e.g., denigratory comments) instead of pretending they do not affect how a consultation is experienced.

#### End of Training: *Setting intentions for practice*

By the end of the IPCT, after the role-play exercises—in which participants experimented with the proposed strategies and reflected on their effects—focus had moved towards a deeper understanding of attitude-related (A) intrapersonal processes and intraprofessional dynamics, with a more collaborative approach observed in consultations. The residents identified specific behaviours, which were rearranged and categorised into three main themes. The key themes describing attitude-driven behaviour were “*affective awareness of themselves, others, and interactions*” and “*collaborative realignment”*. The main theme describing cognitive-driven behaviour was *“shared mental models”* (Table 1, column “*Intention at the end of the training*). In the table, these key themes are illustrated by subthemes, which were most prominently reflected in ‘residents’ intentions during training and applied in their actions in clinical practice. The following paragraph focuses specifically on the training intentions, while actions applied in clinical practice are described in Part 2.

**Table 1:** Intentions at the end of the IPCT and actions in medical practice two months after the IPCT.


	INTENTION IN TRAINING (END OF IPCT)	ACTION IN CLINICAL PRACTICE (REPORTED TWO MONTHS AFTER THE IPCT SESSION)

**Affective awareness**		

Be aware of your own emotions (uncertainty, irritation)	*I just take a few seconds to register how I’m feeling about it [the ‘patient’s problem] and make sure it ‘doesn’t interfere*. (FC4 GP resident)	*At the out-of-hours surgery, I was seeing a child whose parents were very anxious, which made me feel anxious too. As I felt unable to properly separate this feeling from my clinical attitude, I tried to communicate this to the paediatrician in charge*. (QC4 GP resident)

Create mental space for yourself	*When it’s very busy, I attempt to extract myself from the situation to listen properly to the doctor who’s calling*. (FR7 P resident)	*When I was in an examination room assessing a child and a GP called, I asked him/her to please hold the line for a moment so I could finish my examination and leave the room. Colleagues usually don’t mind and it’s much better to be able to talk quietly a little later*. (QR7 P resident)

Check the other person’s mental space	*I ask if it’s convenient for the other person to listen to the story*. (FR5 GP resident)	*As soon as I’m speaking to somebody [the consultant] on the phone, I will mention the reason I’m calling and check first whether they have time. I didn’t check these things explicitly before*. (QC3 GP resident)

Reflect on consultation dynamics	*I intend to reflect more on formal issues and work contacts: what’s fine and what isn’t*. (FR5 P resident)	*I gained a better understanding of “the other side of the line”. They’re not just busy and short-staffed or short of beds, but each time they also need to make an assessment that’s based on the information you are supplying. This is a lot harder on the phone than in a physical consultation [with a patient]. This has changed my way of working in the sense that I now tend to enter into a conversation not so much with the intention and drive of having to convince someone, but rather aiming to inform the other person properly and to come up with a plan together that’s the best for the patient*. (QJ3 GP resident)

**Shared mental models**		

Describe the context in which you are situated to the other person	*Describe the context:* *where I’m sitting during the phone call (“opposite child and parents”)*. (FR2 GP resident)	*Whenever I’m talking to a paediatrician or trainee doctor, I tell them where I am (out-of-hours surgery or elsewhere) and I tell them that patient and parents are sitting opposite me at that moment*. (QR3 GP resident)

Formulate a clear referral question	*Before I make the call, I set out the case, define my question and then enter the conversation*. (FR1 GP resident)	*If you open the consultation with a clear wish (“I would like to refer a patient or consult you”), you notice straightaway that the paediatrician will listen attentively and specifically*. (QR2 GP resident)

Clarify and verify concerns	*Listen carefully and pay attention to the GP’s concerns. “Am I right in thinking your concerns are in …?” “What are your worries?”* (FR3 P resident)	*The action I’ve taken is that I now tend to say out loud: “so would I be right that you’re thinking such and such and that you’re asking/expecting me to do this and that, or do I get you wrong?” This has helped to foster clarity and mutual respect*. (QJ1 P resident)

Explain the rationale	*Say clearly what it is you want from the other person and why*. (FC8 GP resident)	*I’d like to ask a few more questions to clarify the urgency and the option of having the patient come in by private transport*. (QC1 P resident)

**Collaborative realignment**		

Articulate explicitly what you need if consultations are difficult	*If the conversation is tense, mention what your need is*. (C1 GP resident)	*I had a telephone conversation with a GP who was clearly worried … It was utter chaos, which made it really difficult for me to assess the situation. I then said that I understood that the GP was clearly worried, told him that things were still not entirely clear to me, and asked him to summarise the case in a few sentences. He then produced a concise outline of the case, which enabled me to decide that we had to see this child*. (QC4 P resident)

Show understanding for the other person’s position or statement	*[when things are tense] level up, voice your thoughts, and show understanding for his/her situation*. (FJ1 P resident)	*The other day I had a phone conversation that was tense because the specialist saw no grounds for a patient to be referred to him. I showed understanding. Then I said that I heard and understood that this was not an ideal referral, told him I was at my wits’ end, and asked him if he could offer me any alternatives. This helped to open up the conversation*. (QR3 GP resident)

In case of conflict, emphasise the shared goal	*In case of conflict, emphasise the importance of collaboration. You’re phoning up to achieve a common goal. This may help to get everyone to pull together*. (FR4 GP resident)	*A conflict arose on the phone. My request was clear (admission of a patient), and then I attempted to reach out in this conflict. I mentioned the tensions between us, underlining that mine were due to my worries about the patient. I could tell that this helped to ease the strain and that we got a little more air and understanding into the conversation*. (QJ3 GP resident)


***Affective awareness*.** Based on their reflections, residents intended to become more affectively aware of themselves, others, and their interactions, and to acknowledge emotions (see Table 1, *Affective Awareness*). In terms of self-awareness, acknowledging one’s emotions emerged as a key insight. Whilst emotions were initially disengaged, by the end of the IPCT, residents acknowledged feelings such as irritation stemming from feeling patronised or ongoing ambiguity during consultations. They recognised how emotions could impact the consultation process and should be addressed rather than ignored. Mental availability appeared to be crucial in this process. Residents noted that a high workload hindered openness and active listening, and they realised they could influence these factors by creating more mental space for themselves.

Furthermore, residents aimed to be more sensitive to interaction dynamics in intraprofessional consultations and gained deeper insights into the backgrounds of their own and each ‘other’s perspectives and attitudes. This increase in affective awareness became particularly evident in the later stages of the IPCT, following group reflections and the role-play exercise.

***Shared mental models***. In the cognitive process of exchanging information, residents intended to openly discuss their thoughts on the medical content to actively engage the other party and facilitate a clearer shared mental model (see Table 1, *shared mental models*). They acknowledged that they should avoid assuming what others thought and recognised that what seemed logical to themselves might not be so to others. This led to intentions such as explaining the rationale behind certain medical questions.

***Collaborative realignment***. Residents expressed intentions to address and restore tense dynamics in interactions (see Table 1 *collaborative realignment*). In addition to their initial actions of proposing interventions to clarify medical exchange during faltering consultations, residents also planned to engage in metacommunication to restore intraprofessional relationships: for instance, first to align with and acknowledge the other person’s resistance before articulating their own needs. Additionally, residents noted that using terms like “together” or “shared goal” could help to restore the consultation and realign the team.

### Part 2: Clinical Practice — Two-Month Follow-Up of Teamwork ABC Experiences

#### From Intention to Action: *How residents applied their intentions in clinical practice*

Two months after the IPCT training, we examined whether and how intentions were translated into actions in medical practice. Behavioural changes were reported in all three categories (Table 1, column “Action in clinical practice”). Residents primarily applied enhancing affective awareness and optimising shared mental models. Applying behaviour related to collaborative realignment, however, proved more challenging. Residents reported they applied their training intentions not only in consultations between GPs and Ps but also in interactions with other hospital specialists and supervisors.

***Affective awareness***. Regarding affective awareness, residents reported that reflecting on personal emotions, as when they struggled to distinguish their own uncertainty about a child’s illness from that of its parents, created an opportunity for shared analysis (see Table 1, *Affective awareness*). They learned that expressing uncertainty was not a sign of “weakness” but rather a powerful and supportive approach. Residents noted that creating mental space for themselves and others was relatively easy to apply, often with a significant effect, resulting in a sense of calm and clarity in themselves and appreciation from others. They reported a better understanding of others’ positions and contexts, leading to greater acknowledgement and, at times, deeper reflection and adjustments in their own attitudes and beliefs.

***Shared mental models*.** Actions related to optimising shared mental models, such as a clear handover structure, were frequently described (see Table 1, *Shared mental models*). Residents reported that they had a clearer understanding of their own and others’ concerns, identifying opportunities to support each other and optimise shared decision-making.

***Collaborative realignment***. With regard to collaborative realignment, residents reported behaviour that involved briefly acknowledging tension, followed by clarifying their own perspective or demonstrating understanding of the other ‘person’s point of view (see Table 1, *Collaborative realignment*). Efforts were then made to move from tension to shared purpose by regaining alignment and emphasising the importance of collaboration for the patient. Residents described they applied their intention to realign a difficult consultation less frequently than proposed. Specifically, intentions such as openly signaling and engaging in dialogue about difficulties in the collaborative process, sharing reflections, concerns, and needs regarding the collaborative relationship, or inquiring how the other person experienced the collaboration were rarely applied in practice.

#### Barriers to Translating Intentions into Action: *Challenges Perceived by Residents*

Residents indicated that, although they recognised tensions in intraprofessional dynamics, they still felt reluctant to articulate them and, particularly, to engage in dialogue about them.

*I’m more aware now of the telephone conversations I conduct, and so I’m also more aware of what gives rise to frustrations or bottlenecks in these conversations. The next step for me will be to voice these things distinctly in the conversation*.

QC4 P resident

The rigid boundaries between specialties were perceived as barriers to engaging in collaborative problem-solving when the issue fell outside one’s area of expertise. Residents also reported it was challenging to openly discuss friction when time was pressing and when they were in a dependent position.

*When things are not going smoothly, it’s always hard to point this out because I usually need something from the other person. I should realise though that the other person also needs something from me*.

QJ1 GP resident

Residents identified the presence of the child’s parents in the room as a barrier to openly expressing uncertainty during a consultation.

*I thought this was pretty difficult [to show uncertainty about the case to the P resident] because the parents were present and I didn’t want to come across as unprofessional or ignorant [to the parents]*.

QC4 GP resident

## Discussion

In our research, we found that P and GP residents primarily developed insights into attitudes and affective states associated with teamwork. In clinical practice, they applied lessons learned to enhance affective awareness of themselves, others, and intraprofessional interactions. This awareness enabled them to acknowledge rather than ignore emotions, foster a more open mindset, and create greater mental space. Additionally, residents gained an understanding of how to optimise shared mental models when anticipating or addressing potential ambiguities during consultations. Residents also achieved a deeper understanding of the dynamics underlying tense intraprofessional consultations. Applying these skills to address such dynamics in medical practice, however, remains a significant challenge.

Given the observed learning impact on affective and motivational states that contribute to team performance dynamics, and the persistent challenge of managing difficult collaboration dynamics in practice, we will now discuss the capacity to remain connected to oneself, others, and the team under stressful conditions and the potential reasons that make navigating collaborative dynamics in clinical practice complex.

### Maintaining connection in acute medical care

In our study, residents reported key insights into becoming more affectively aware of themselves, others, and interactions, as well as acknowledging their emotions. This awareness improved their ability to maintain contact with themselves and others, and to analyse intraprofessional dynamics. As these outcomes extend beyond (self-)reflection in action, the findings led us to seek a more comprehensive concept to cover them [27,28,29]. Mentalising, a concept from the field of psychiatry [30,31], refers to the ability to interpret both one’s own behaviour and that of others in terms of mental states, such as emotions and beliefs. It consists of the interdependent triad of self-reflection, empathy, and interactional interpretation in order to discern and modify emotional, relational, and communicative processes while interacting with others [31].

Learning to mentalise under stressful circumstances is particularly important as stress can impair mentalising capacity and disrupt teamwork [32,33]. This means team members may appear to participate but are mentally disengaged, or conversely, become emotionally overwhelmed, which reduces their ability to accurately understand others’ perspectives [34,35,36,37]. Both disengagement and emotional overwhelm hinder meaningful interaction and increase the risk of misunderstandings. Consequently, the ability to co-construct a shared mental model regarding a patient’s situation—achieving a common understanding and approach from initially differing perspectives—declines when mentalising capacity is compromised. This, in turn, limits opportunities for shared decision-making and increases the likelihood of ill-informed decisions [7,32,38,39]. Effective mentalising supports the development and maintenance of shared mental models by enabling team members to remain open to each other’s thoughts and intentions, and to adjust their own understanding accordingly, thereby fostering shared decision-making.

Evidence from psychiatry indicates that mentalising and recognising disruptions in mentalising capacity are trainable skills [30,34], which may have emerged as an unintended effect of our IPCT sessions [29]. Group reflection and role-playing exercises, incorporating attention to perceived emotional distance and proximity, appeared particularly valuable for fostering the development of mentalising skills. These insights provide a foundation for further development in medical education, particularly supporting the learning of teamwork skills in high-pressure collaborative settings [33,39].

### Emotional dynamics in collaboration: challenges in clinical practice

Despite this increase in affective awareness, our findings indicate that openly addressing the emotional dimensions of collaboration in clinical practice remains challenging. One important phenomenon that warrants closer attention in this regard is the emotional socialisation of physicians: during medical training, doctors are taught to shield themselves from emotions—both as a coping mechanism to manage patients’ suffering and as a professional norm [40]. While this defensive stance may offer protection, it can also impede opportunities for mutual support in collaborative work and may promote emotional distance and conflict avoidance when tensions arise in the collaborative relationship [41,42]. Our results showed that residents were aware of emotional dynamics in collaboration, even early during the IPCT (Figure 3), yet they tended to keep these experiences to themselves, relying on alternative strategies rather than openly sharing feelings of uncertainty or intraprofessional tension. With encouragement from training instructors and in a context that actively stimulates open dialogue, residents appeared more willing to propose and discuss alternative approaches. Thus, creating an open setting and providing explicit encouragement from instructors and supervisors seems essential to surface strategies that may already exist but remain hidden within professionals, as well as to cultivate new ones—especially given that reaching collaborative realignment is inherently complex.

At the same time, hierarchical structures and professional dependency appear to reinforce avoidance in clinical practice [43]. Residents indicated that addressing tensions during consultations felt risky, as doing so depended not only on their own confidence but also on the willingness of others to engage in dialogue. In such dynamics, hierarchy can exacerbate hesitation, making silence or redirection the more pragmatic option [16,43].

Our findings suggest that supporting residents in expressing the emotional aspects of professional and collaborative relationships—and experiencing its constructive effects—can be an important step toward change. To sustain this shift, supervisors play a pivotal role by role-modelling respectful, open, and vulnerable collaboration. Yet given that the same processes of emotional socialisation have shaped supervisors themselves, this represents not only an individual challenge but also a broader cultural transition in medicine.

### Strengths and limitations

The training content was developed by the research group, potentially introducing bias when analysing the impact of the IPCT. To mitigate this, investigator triangulation was employed, involving a multidisciplinary team with expertise in postgraduate medical education, clinical practice, and educational research, ensuring diverse perspectives in data analysis. Additionally, aggregating data sources generated during and after the IPCT enabled the research team to study what actually occurred, as well as what participants self-reported. By following up on how residents applied their intended strategies in real-world settings, the study provided valuable insights into barriers to application and directions for future research. This longitudinal design was therefore particularly valuable, consistent with the emphasis on longitudinal qualitative approaches in medical education [44]. A potential limitation is the voluntary participation of residents, which may have introduced selection bias, potentially overrepresenting more motivated individuals. For the residents, the IPCT was part of a broader intraprofessional educational programme. Nevertheless, by deliberately using a clear analytical lens based on the ABC model for template development, the results could be analysed with reasonable precision, reducing the risk that the findings were driven by participation in the larger program. Although this clear theoretical lens guided the template evolution process, it also allowed for an inductive exploration of additional emerging patterns. The involvement of a heterogeneous research team reviewing the data and a psychiatrist reflecting on the findings helped prevent a deductive tunnel vision. To enhance the transferability of our findings, participants from different non-academic teaching hospitals were included. Finally, a comprehensive description of the IPCT is provided, to support understanding of the research context.

### Implications for practice and future research

The capacity for mentalisation and its potential impact on teamwork in high-pressure collaborative settings represent a promising avenue for further research in medical education. More evidence is needed to determine whether, and how, the development of mentalising skills can be attributed to designed training. Future studies should also explore interventions that help healthcare professionals address emotional processes and teamwork dynamics in clinical practice. Supervisors play a crucial role in modelling such behaviours, yet they may also need guidance, as engaging with emotional (team) dynamics is not yet a cultural norm in medical practice.

## Conclusion

Our research demonstrated that GP and P residents primarily developed insights and strategies related to attitudes and emotions influencing teamwork behaviour. Residents became more aware of their own emotions, those of others, and the emotional dynamics during interactions, aligning with the concept of mentalising. The training fostered shared mental models and supported interventions to create clarity and a shared understanding. Navigating and resolving tense and complex collaborative dynamics in clinical practice remains challenging. Sustainable integration into medical practice requires not only that professionals manage intraprofessional dynamics individually, but also that recognition and handling of these dynamics become embedded in everyday work culture. In doing so, healthcare professionals can actively provide mutual emotional support and leverage each other’s expertise in a high-demand environment, benefiting themselves, their colleagues, and their patients.

## Additional Files

The additional files for this article can be found as follows:

10.5334/pme.1770.s1 Appendix A. Initial and final template.

10.5334/pme.1770.s2 Appendix B. Transcript of Intraprofessional Consultation Video Scenario between General Practitioner and Paediatrician.

10.5334/pme.1770.s3 Appendix C. Appendix C: Post-intraprofessional consultation (ICT) questionnaire.
