# Supplementary material for: Unveiling Intraprofessional Dynamics: Learning Teamwork in Acute Care Consultations Between Paediatric and General Practice Residents
Source: Perspect Med Educ. 2025 Nov 13;14(1):800–12. doi: 10.5334/pme.1770 (PMC12617421; doi:10.5334/pme.1770)
Supplement: Appendix A. — Initial and final template. [file pme-14-1-1770-s1.pdf]

Appendix A: Initial and final template

Initial template

The comprehensive framework of attitudes (A), behaviours (B), and cognitions (C) of teamwork served as an initial template. The initial template was rearranged around the concepts of attitude-driven behaviour (behaviour in which feelings, perceptions, and principles about oneself and/or relationships with others are acknowledged, expressed, or discussed) and cognition-driven behaviour (behaviour based on cognitive processes involved in acquiring, processing, and structuring (medical) knowledge and information), incorporating the following a priori themes:

Attitude-driven behaviour:

- mood/emotions
- trust
- respect
- hierarchy
- intervention in tense dynamics.

Cognition-driven behaviour:

- shared mental model (subdivided into "shared goals", shared perception", and "shared understanding of team roles")
- shared decision-making.

The theme of "context to apply intentions" was included as an a priori code to examine where learned insights were reactivated and applied.

Development

The key themes describing attitude-driven behaviour were "*affective awareness of oneself, others, and interactions*" and "*collaborative realignment"*. The main theme describing cognitive-driven behaviour was *"shared mental models".* Three sub-themes ("professional attitude", "mental availability", and "awareness/acknowledgment of intraprofessional dynamics") were added to the first attitude-driven behaviour theme. The main theme describing cognitive-driven behaviour was "shared mental models". Regarding transfer of intentions into actions, "barriers to applying intentions in practice" was added to the template. The final template, incorporating all identified topics, was structured as follows:

Final template

<u>Attitude-driven behaviour</u> (Ab)

Main theme 1: Affective awareness of themselves/others/interactions

Themes and codes

1. **Mood/emotions**

1.1 Acknowledging your own emotions (feeling uncertain/irritated/patronized/confused/angry)

1.2 Actively monitoring the other person's emotions (sounds worried/overwhelmed/irritated)

2. **Professional attitude: stance/tone**

2.1 Remaining friendly (avoid being affected by hectic situations or personal stress)

2.1.1 Friendly intonation

2.2 Open attitude

2.3 Neutral stance

2.4 Refraining from displaying irritation

2.5 Refraining from criticizing the other professional personally

2.6 Not projecting your own issues onto others

3. **Mental availability**

3.1 Your own mental space

3.1.1 Awareness of your own mental space

3.1.2 Creating more mental space

3.2 Mental space of the other person

3.2.1 Monitoring the other person's mental space

3.2.2 Actively checking the other person's mental space

4. **Awareness/acknowledging intraprofessional consultation dynamics**

4.1 Empathy and understanding

4.1.1 Feeling understanding of your own position/perspective

4.1.2 Gaining understanding of the other person's position/perspective

4.1.3 Expressing understanding of the other person's situation

4.2 Understanding the background of consultation dynamics

4.2.1 Understanding the impact of workload on the consultation dynamics

4.2.2 Understanding the impact of the other person's context on consultation dynamics

4.3 Recognizing tension in consultation dynamics

4.3.1 Recognizing, pausing, and trying to really focus on what you are feeling

4.3.2 Recognizing and trying to reflect on its origin

5. **Trust**

5.1 Openly expressing doubt

5.2 Openly expressing uncertainty

5.3 Not using improper reasoning

6. **Respect**

6.1 The importance of feeling respected (position)

6.2 The importance of genuinely respecting the other person's position

6.3 Giving feedback in a respectful manner

7. **Hierarchy**

7.1 (Feeling) the impact of hierarchy/dependency on consultation dynamics

Main theme 2: Collaborative realignment

8. **Intervention in tense consultation dynamics (conflict)**

8.1 During friction in consultations: articulating explicitly what you need

8.2 Showing understanding for the other person's position/statement

8.3 In case of friction: emphasizing the shared goal

8.3.1 By using the words "together" or "shared"

8.3.2 Emphasizing the importance of collaboration for the benefit of the patient

8.3.3 Highlighting the commonalities (feelings/thoughts) between the two different contexts

8.4 In the event of friction: focusing on rebuilding relationship (dialogue) instead of focusing on medical content

8.4.1 Naming what you believe you are perceiving in the other person and engaging in a dialogue about it

8.4.2 Offering a reflection on what you are experiencing yourself and initiating a dialogue from there

8.4.3 Explicitly pausing the intraprofessional consultation

Cognition-driven behaviour (Cb)

Main theme 3: Shared mental models

9. **Optimizing shared mental models**

Shared goal:

9.1 Clear referral question and hand-over structure

9.2 Stating clear referral question at start of consultation

9.3 Actively checking referral question

9.4 Using a clear handover structure (SBAR), including a structured presentation of vital parameters

9.5 Summarizing (the key findings and the reason for consultation)

Shared perception:

9.6 Describing the context in which you are situated to the other person (mention context/workload)

9.7 Clarifying concerns to the other person

9.8 Verifying concerns of the other person

9.9 Distinguishing between the concerns of the professional and those of the patient/parent

9.10 Mentioning explicitly if parental/patient concern is a key factor in the consultation/referral

9.11 Explaining or exploring the rationale (behind question/statements/medical policy)

Shared understanding of team roles

9.12 Understanding the other person's context and adjusting expectations accordingly

9.13 Understanding the other person's role and adjusting expectations accordingly

10. **Shared decision-making**

10.1 Aligning policies/procedures

10.2 Expectation management in referrals

10.3 Agreement on safety net advice

<u>Other</u>

11. **Context to apply intentions**

11.1 IP consultations between GP and paediatricians

11.2 IP consultations with other acute care physicians

11.3 IP consultations with other (non-acute) care physicians

11.4 In collaboration with others (other health care professionals or between resident-supervisors)

12. **Barriers to application of intentions**

12.1 Finding it difficult/challenging to engage in dialogue about a tense collaborative relationship

12.2 Lack of time

12.3 Feeling dependent on the other person

12.4 Patient/parents in consultation room
