# Supplementary material for: Unveiling Intraprofessional Dynamics: Learning Teamwork in Acute Care Consultations Between Paediatric and General Practice Residents
Source: Perspect Med Educ. 2025 Nov 13;14(1):800–12. doi: 10.5334/pme.1770 (PMC12617421; doi:10.5334/pme.1770)
Supplement: Appendix B. — Transcript of Intraprofessional Consultation Video Scenario between General Practitioner and Paediatrician. [file pme-14-1-1770-s2.pdf]

Appendix B Transcript of Intraprofessional Consultation Video Scenario between General Practitioner and Paediatrician

P = Paediatrician, GP = General Practitioner

[Phone rings]

P: Yes, Wiggelaar speaking.

GP: Good evening, this is Susan van Berkel, general practitioner. I'd like to discuss something with you.

P: Erm, yes, if it's brief, because I have a handover shortly.

GP: Oh, I'll try to be quick. I'm seeing an 18-month-old girl with no medical history. Vaccinated as per schedule, and has had persistent high fever for the past three days. She's drinking only half of what she normally does, but still has wet diapers. Now she's breathing faster, a bit lethargic, and I'm noticing spots on her skin.

P: [Interrupts GP] If I may interrupt you for a moment: if this is about a potential admission, we currently don't have any beds available at the hospital.

GP: I heard about that, but I'd still like to finish explaining the case.

P: Yes, go ahead.

GP: It's a young girl with high fever, faster breathing, and some lethargy. The parents also mention that she's not as alert as usual. She's getting worse rather than better. So I thought it might be wise for you to take a look.

P: Well, from what you're telling me, I'm not entirely convinced that I need to see this child. There are so many children right now with faster breathing and fever. We're in the middle of the RSV season.

GP: Yes, I'm aware of that. I've seen a lot of cases like that too. But you know, the parents are extremely worried. Their friends' daughter was recently diagnosed with meningitis, so they're afraid their child might have the same. There hasn't been any contact between the children, but still. I've thoroughly examined this child and conducted some additional tests here at the practice, but I can't alleviate their concerns.

P: Yes, well, of course, that's very unfortunate for those parents. But we're not here to admit children just to calm down anxious parents, are we? I don't currently see any medical necessity.

GP: I understand that as well. I'm not here to refer every child of worried parents. You wouldn't believe how many children I've seen tonight and sent home responsibly. But this case is a bit more complex, and your evaluation would really help. It would reassure the parents, and me as well, and we could definitively rule out meningitis.

P: Yes, yes. But is that your actual question? Are you doubting your diagnosis? Because based on what you've told me, it doesn't seem very logical to doubt it.

GP: No, but exceptions always exist. Recently, I had a case with a boy presenting with abdominal pain. Turned out to be appendicitis. Not logical, but there it was.

P: [Interrupts GP] Okay, just send her in then. This conversation is already taking too long anyway. So tell the parents I'll see their daughter, but they should know that it's extremely busy in the emergency department, and the waiting time will be long. If the child does need to be admitted, she'll have to be transferred because we have no beds. And make sure she gets adequate paracetamol beforehand.

GP: Yes, we've already given that, but it only works for about an hour. Anyway, I'm looking forward to your assessment, and I'll discuss it with the parents.

P: [Talking over GP] Good, fine, fine. Alright, goodbye.

GP: [Talking over P] Thank you, goodbye.
