# Supplementary material for: Unveiling Intraprofessional Dynamics: Learning Teamwork in Acute Care Consultations Between Paediatric and General Practice Residents
Source: Perspect Med Educ. 2025 Nov 13;14(1):800–12. doi: 10.5334/pme.1770 (PMC12617421; doi:10.5334/pme.1770)
Supplement: Appendix C. — Appendix C: Post-intraprofessional consultation (ICT) questionnaire. [file pme-14-1-1770-s3.pdf]

Appendix C: Post-intraprofessional consultation (ICT) questionnaire:

Dear Resident,

On ………, you participated in the training:

The ABC of Intraprofessional Consultation in Acute Paediatric Care.

The aim of this training was to enhance collaboration skills (behaviour) in managing acutely ill children by exploring and explicating attitudes (feelings, perspectives) and cognitions (thoughts) relating to complex consultations between general practitioners and paediatricians.

As part of the research associated with this training, we would like to ask you a few questions. These questions aim to assess the extent to which the training has supported you during intraprofessional consultations (consultations between medical colleagues within the same discipline) in practice. Completing the questionnaire will take approximately 15 minutes.

As explained in the information letter, the questionnaire will be pseudonymized. When we receive your completed questionnaire, a pseudonym will be assigned, and your name will be removed, ensuring that your responses cannot be directly traced back to you. The pseudonym and your name will be stored in a key file on the secure network drive of the Department of Primary Care at Radboudumc, accessible only to the principal investigator.

Thank you again for your participation and contribution to this research.

Kind regards,

Could you try to answer the questions below as thoroughly as possible

Appendix C: Post-intraprofessional consultation (ICT) questionnaire:

Question 1:

Are you a resident in General Practice or a resident in Paediatrics?

Question 2:

What key insight from the training has stayed with you?

Question 3:

To what extent has this insight influenced your perspective or way of working?

Question 4:

At the end of the training, you wrote down two intentions for things you wanted to do differently or more often during intraprofessional consultations in practice.

1)

2)

What were these intentions?

Appendix C: Post-intraprofessional consultation (ICT) questionnaire:

Question 5:

Which of these intentions have you been able to apply in practice? Answer only for those you have implemented.

Question 6:

Can you describe one or two examples of how you applied your intention(s)?

Question 7:

In what setting did you apply these intention(s)? Was it during consultations between general practice and paediatrics, or also during consultations with other colleagues?

Question 8:

In general: Has participating in the training helped you during (challenging) consultations with colleagues in practice?

Yes/No (Circle your answer)

If yes: How has participating in the training helped you?

Question 9:

May we contact you for further clarification of your responses in an interview?

Appendix C: Post-intraprofessional consultation (ICT) questionnaire:

Yes/No (Circle your answer)
